# BARIATRIC SURGERY & SUICIDE: RESULTS FROM TWO CONTROLLED MATCHED COHORT STUDIES

**DOI:** 10.1016/S2213-8587(17)30437-0

**Published:** 2018-01-09

**Authors:** Martin Neovius, Gustaf Bruze, Peter Jacobson, Kajsa Sjöholm, Kari Johansson, Fredrik Granath, Johan Sundström, Ingmar Näslund, Claude Marcus, Johan Ottosson, Markku Peltonen, Lena M.S. Carlsson

**Affiliations:** 1 Department of Medicine, Solna, Clinical Epidemiology Unit, Karolinska Institutet, Stockholm, Sweden; 2 Institute of Medicine, The Sahlgrenska Academy, University of Gothenburg, Gothenburg, Sweden; 3 Department of Medical Sciences, Uppsala University, Uppsala, Sweden; 4 Department of Surgery, Faculty of Medicine and Health, Örebro University, Örebro, Sweden; 5 Department of Clinical Science, Intervention and Technology, Karolinska Institutet, Stockholm, Sweden; 6 Department of Chronic Disease Prevention, National Institute for Health and Welfare, Helsinki, Finland

## Abstract

**BACKGROUND:**

Bariatric surgery reduces mortality, but may have adverse effects on mental health. We assessed suicide risk after surgical compared to nonsurgical obesity treatment.

**METHODS:**

Suicide and nonfatal self-harm events retrieved from nationwide Swedish registers were examined in two cohorts. The nonrandomised prospective Swedish Obese Subjects (SOS) study compares bariatric surgery (n=2010; 1369 vertical-banded gastroplasty, 376 gastric banding, 265 gastric bypass) with usual care (n=2037; recruitment 1987–2001). The second cohort comprises individuals from the Scandinavian Obesity Surgery Registry (SOReg; n=20,256 gastric bypass patients) matched to individuals treated with intensive lifestyle modification (n=16,162; intervention 2006–2013) on baseline BMI, age, sex, education level, diabetes, cardiovascular disease, history of self-harm, substance abuse, antidepressant use, anxiolytics use, and psychiatric healthcare contacts.

**FINDINGS:**

During 68,528 person-years (median 18; interquartile range 14–21) in SOS, there were 87 versus 49 suicides or nonfatal self-harm events in the surgery and control groups (adjusted hazard ratio [aHR] 1.78 [95%CI 1.23–2.57]; P=0.0021), of which 9 and 3 were suicides (3.06 [0.79–11.9]; P=0.107). In analyses by primary procedure type, increased risk of suicide or nonfatal self-harm was observed for gastric bypass (aHR 3.48 *1.65–7.31+; P=0.0010), gastric banding (2.43 *1.23–4.82+; P=0.011) and vertical-banded gastroplasty compared to controls (2.25 *1.37–3.71+; P=0.0015). Out of 9 deaths by suicide in the SOS surgery group, 5 occurred after gastric bypass (2 primary and 3 converted procedures). During 149,582 person-years (median 3.9; interquartile range 2.8–5.2), there were 341 suicides or nonfatal self-harm events in the SOReg gastric bypass group and 84 in the intensive lifestyle group (aHR 3.16 [2.46–4.06]; P<0.0001), of which 33 and 5 were suicides (5.17 [1.86–14.4]; P=0.0017). In SOS, substance abuse was recorded in 48% (39/81) of surgery patients and 28% (13/47) of controls with nonfatal self-harm events (P=0.023). The corresponding percentages for SOReg gastric bypass and intensive lifestyle participants were 51% (162/316) versus 29% (23/80; P=0.0003).

**INTERPRETATION:**

Bariatric surgery was associated with suicide and nonfatal self-harm. Although the absolute risks were low, the findings indicate a need for post-operative psychiatric surveillance and patient information before surgery regarding self-harm.

**FUNDING:**

US National Institutes of Health and Swedish Research Council

## INTRODUCTION

In 2014, an estimated 125 million women (5.0%) and 50 million men (2.3%) globally had a BMI≥35kg/m^2^,(1) making them potentially eligible for bariatric surgery. Bariatric surgery reduces the risk of premature death,(2–4) cardiovascular events,(5, 6) and micro-/macro-vascular diabetes complications.(7, 8) However, there is growing concern about adverse effects on mental health, with increased alcohol and substance abuse after some procedures, as well as signals of an increased suicide risk compared with morbidly obese individuals.(9, 10) Compared with the general population, bariatric surgery patients have been reported to have higher risk of both suicide(11, 12) and nonfatal self-harm.(13) Nonfatal self-harm events are also more common after than before surgery.(12–15)

As suicide is rare, it is unlikely that there will ever be a randomised trial of sufficient size and duration to assess suicide risk after bariatric surgery. Further, there are no observational studies on suicide comparing bariatric surgery patients with nonsurgically treated obese controls. The Utah Mortality Study(2) reported an increased risk of “deaths not caused by disease” in patients treated with bariatric surgery compared to age-sex-BMI-matched controls applying for a driver’s licence. The risk of suicide was not statistically significant, but the point estimate was more than twice as high in surgery patients compared to matched controls. In the Utah Obesity Study,(16) no difference in suicide risk over up to 6 years could be detected in the bariatric surgery group (4 suicides) versus a morbidly obese control group seeking but not receiving bariatric surgery (0 suicides). Neither study accounted for baseline psychiatric status (which is likely to be associated with both bariatric surgery exposure and the outcome suicide) between the surgery and control group, nor had they a nonsurgically treated obese control group. A recent Danish cohort study excluded patients with history of psychiatric contacts and reported no difference in suicide rates between bariatric surgery patients versus hospitalised patients with a diagnosis of obesity but without bariatric surgery.(15)

We aimed to compare the risk of suicide and nonfatal self-harm in patients with obesity attempting to lose weight with versus without bariatric surgery, accounting for baseline psychiatric status in two Swedish matched cohort studies linked to outcome data from nationwide health registers.

## METHODS

### Study Design

Matched cohort designs were used to analyse the association between bariatric surgery and the outcomes suicide and nonfatal self-harm. The cohorts used for the current analysis were the Swedish Obese Subjects (SOS) study(3) and a nationwide register linkage combining the Scandinavian Obesity Surgery Registry (SOReg)(17) with the Itrim Health Database, a register including individuals treated with intensive lifestyle modification.(6) The rationale for using two studies was that SOS and SOReg/Itrim have complementary strengths. SOS provides longer follow-up than any other existing controlled study, but used older surgical techniques. SOReg/Itrim included current surgical techniques and an intensively treated control group, but had shorter follow-up.

SOS and SOReg/Itrim participants were linked to nationwide health registers using the Swedish personal identity number which is unique for each resident. The linkage was performed by officials at the National Board of Health and Welfare and at Statistics Sweden in 2015 and 2016.

### Setting

The Swedish health care system is tax funded and offers universal access, including physicians, psychologists, dietitians and other healthcare specialists. The adult prevalence of BMI≥35kg/m^2^ in Sweden in 2014 has been estimated to 5–6%.(1) In a global perspective, Sweden had one of the highest percentages of bariatric procedures for the total population in 2013 (0.08% as compared with 0.04% in the US and Canada).(18) In individuals undergoing bariatric surgery, the prevalence of depression, self-harm, and substance abuse at baseline is about twice as high as in the general population in Sweden.(13) The suicide rate in Sweden is similar to the OECD average and that in the United States (12.3, 12.0 and 12.5 per 100,000.(19)

### The SOS Study

This prospective, nonrandomised, controlled intervention study recruited patients from September 1, 1987, to January 31, 2001(3) via recruitment campaigns in the mass media and at 480 primary healthcare centers. Patients choosing surgery constituted the surgery group. From individuals not choosing surgery, a contemporaneously matched control group was created using 18 matching variables: sex, age, weight, height, waist circumference, hip circumference, systolic blood pressure, serum cholesterol and triglyceride levels, smoking status, diabetes, menopausal status, 4 psychosocial variables with documented associations with death, and 2 personality traits related to treatment preference (data on psychosocial variables and personality traits are provided in eTable1). Matching was not performed at an individual level but an algorithm selected controls so that the current mean values of the matching variables in the control group became as similar as possible to those in the surgery group using the method of sequential treatment assignment.

#### Inclusion/exclusion criteria

Study groups had identical inclusion (age 37–60y and BMI≥34kg/m^2^ in men and ≥38 kg/m^2^ in women) and exclusion criteria (earlier surgery for gastric or duodenal ulcer, earlier bariatric surgery, gastric ulcer or myocardial infarction during the past 6 months, ongoing or active malignancy during the past 5 years, bulimic eating pattern, drug or alcohol abuse, psychiatric or cooperative problems contraindicating bariatric surgery, and other contraindicating conditions such as chronic glucocorticoid or anti-inflammatory treatment).

#### Interventions

The choice of procedure was made by the operating surgeon (265 [13%] gastric bypass, 376 [19%] gastric banding, 1369 [68%] vertical-banded gastroplasty). Open surgery was used in 89% of the patients. Laparoscopic surgery was gradually introduced from 1993 and during the last 2 recruitment years the majority of procedures were performed using this technique. Control patients received the customary nonsurgical obesity treatment at their registration center. No attempt was made to standardise the nonsurgical treatment, which ranged from sophisticated lifestyle intervention to no treatment.

### The SOReg/Itrim Study

SOReg is a nationwide, prospective register for bariatric surgery started in 2007. It has been estimated to cover 98.5% of all bariatric procedures in Sweden.(17) Data are stored electronically and recorded as part of clinical practice. For this study, data were used from intervention years 2007 to 2012.

The Itrim Health Database prospectively collects data on individuals who enroll in the commercial weight loss program at 38 Itrim centers across Sweden. Itrim centers use a common IT platform for quarterly follow-up of, for example measured weight, waist circumference, and blood pressure. For this study, data were available from individuals starting the program from January 1, 2006, to December 31, 2013.

#### Inclusion/exclusion criteria

In the current report, individuals ≥18y with BMI 30–49.9kg/m^2^ and baseline weight recorded were included from SOReg and Itrim. There were no mandatory national eligibility criteria for bariatric surgery during the study period, but most county councils recommended BMI≥35 with or BMI≥40kg/m^2^ without obesity-related comorbidity. In the sample used for this study, 888 surgery patients (4.0%) had a BMI<35kg/m^2^ (median BMI: 34.1kg/m^2^).

#### Interventions

Surgery participants underwent primary gastric bypass (96.0% of procedures conducted laparoscopically; open surgery was primarily used when a patient had had a previous open abdominal surgery or when complications arose during an initially laparoscopic procedure).

Intensive lifestyle participants received the Itrim program including a 3-month weight loss phase with either low or very low calorie diets (eMethods) based on baseline BMI, personal preference, and contraindication status. After the weight loss phase, patients entered a 9-month weight maintenance program including exercise (circuit training at the center 2–3 times/week for 30–45 minutes, and pedometer use to encourage walking), and dietary advice. Behavioral changes were facilitated by a structured program, including twenty 1h group sessions. There were also face-to-face counseling sessions throughout the program.

### Covariates in SOS and SOReg/Itrim

Demographic data were available on age, sex, and educational level. For SOReg/Itrim, data were retrieved from Statistics Sweden on marital status, disposable income, disability pension (also available for SOS), and unemployment. Measured BMI was available from baseline examinations. Data on healthcare visits for self-harm, substance abuse, and other psychiatric causes, as well as for cardiovascular disease, were retrieved from the National Patient Register (inpatient data from 1969; hospital-based outpatient data from January 1, 2001). Data on psychiatric and anti-diabetic drug use before inclusion were retrieved via self-report in SOS and from the Prescribed Drug Register in SOReg/Itrim (register start date: July 1, 2005). Self-reported drug use in SOS has previously been shown to be reasonably consistent with data from the Prescribed Drug Register.(20)

The International Classification of Diseases (ICD) and Anatomical Therapeutical Chemical classification system codes used are provided in eTable2. As missing data on BMI (0.02% [12/61,495]) and education (0.4% [254/61,495]) were rare in SOReg/Itrim, and data were complete for the other variables, patients with missing data were excluded.

### Outcome and Follow-Up in SOS and SOReg/Itrim

The primary outcome in SOS was all cause mortality, for which the study was powered.(3) The outcomes of the current analysis were death by suicide, and death by suicide or nonfatal self-harm, retrieved from the Causes of Death Register and the National Patient Register until December 31, 2013, for SOS and December 31, 2014, for SOReg/Itrim. In the main analysis, we used ICD codes to identify suicide and nonfatal self-harm (ICD9: E950-959, E980-989; ICD10 X60-84, Y10-34, Y870), including both confirmed suicides and deaths from undetermined intent.

Participants were followed from the treatment start date until first event, death, emigration, or end of register-based follow-up, whichever came first. SOS controls and Itrim participants who crossed over to bariatric surgery were censored at the cross-over date (SOS n=289; Itrim=335), as were SOS surgery patients who had their procedure reversed to normal anatomy (n=100).

During follow-up, two SOS surgery patients requested to be deleted from the database, and one obtained an unlisted identity number making linkage impossible. In SOS, both groups had identical follow-up with physical examinations and questionnaires at baseline and 0.5, 1, 2, 3, 4, 6, 8, 10, 15 and 20 years. In addition to the follow-up for the research study, SOS patients also had routine follow-up in the public healthcare system (eMethods).

### Statistical Analysis

Outcomes were analysed using survival analysis. Hazard ratios were estimated using Cox regression. The proportional hazard assumption was evaluated by interacting time and treatment. This term was statistically significant for suicide in SOReg/Itrim (P=0.0497). Due to the small number of events, the model was not stratified by follow-up time.

In the sequentially matched SOS study, adjustment was made for age, sex, history of self-harm (yes/no), and continuous BMI. In the current SOReg/Itrim analysis, we used coarsened exact matching(21) to match participants by BMI (<35, 35 to <40, 40 to <45, 45 to <50kg/m^2^), age (18–29, 30–39, 40–49, 50–59, ≥60 years), sex, education level, diabetes, cardiovascular disease, history of self-harm, substance abuse, antidepressant use, anxiolytics use, and history of psychiatric care (yes/no). To minimise loss of information, we allowed matching strata to include different numbers of surgery and intensive lifestyle participants. To compensate for the differential strata sizes, analyses were weighted by the strata size. For example, if there were 2 surgery participants and 4 lifestyle participants in a stratum, then each surgery participant was given the weight 1 and each lifestyle participant the weight 0.5. Additional adjustment was performed for age, BMI, and income as continuous variables, and for marital status (married/unmarried), disability pension (yes/no), and unemployment benefits (yes/no).

Subgroup analyses were performed by procedure type (SOS only; analysis by intention-to-treat), psychiatric history, and education level. In SOS, the 10-year weight trajectory was examined in surgery patients with an event versus those without.

Statistical analyses were performed using SAS (version 9.4) and Stata (version 14). The SOS study is registered with ClinicalTrials.gov NCT01479452.

### Role of the Funding Source

The funders of the study had no role in study design, data collection, data analysis, data interpretation, or writing of the report. MN, GB and LMSC had full access to the data in the study. The corresponding author had final responsibility for the decision to submit for publication.

## RESULTS

After recruitment campaigns in the mass media and at primary healthcare centers, 6905 individuals completed an eligibility examination for the SOS study, 5335 were found eligible of which 2010 chose surgical treatment while the contemporaneously matched control group consisted of 2037 individuals not choosing surgery (eFigure1).

Out of 30,081 SOReg patients who had bariatric surgery during the study period, 26,388 had gastric bypass and were eligible for matching, while 18,365 out of 31,414 intensive lifestyle participants were eligible (eFigure2). After matching, there were 20,256 (77%) gastric bypass and 16,162 (88%) intensive lifestyle participants available for analysis.

Baseline characteristics in the two cohorts are presented in Table 1. SOS patients in the surgery group had lower education, more history of hospitalisation for self-harm and cardiovascular disease, and were younger and had a higher BMI compared to controls. Mean body weight changes in the surgery and control group at 2, 10 and 15 years were −23%/0%, −17%/1% and −16%/−1%, respectively.

In the SOReg/Itrim cohort, the prevalence of class I, II and III obesity was identical after matching but gastric bypass patients had a higher mean BMI than intensive lifestyle participants. Gastric bypass patients also had lower income, were more often married, on disability pension, unemployed, and using hypnotics or sedatives. The mean 1-year body weight change was −32% in the gastric bypass and −15% in the intensive lifestyle group.

**Figure 1:**
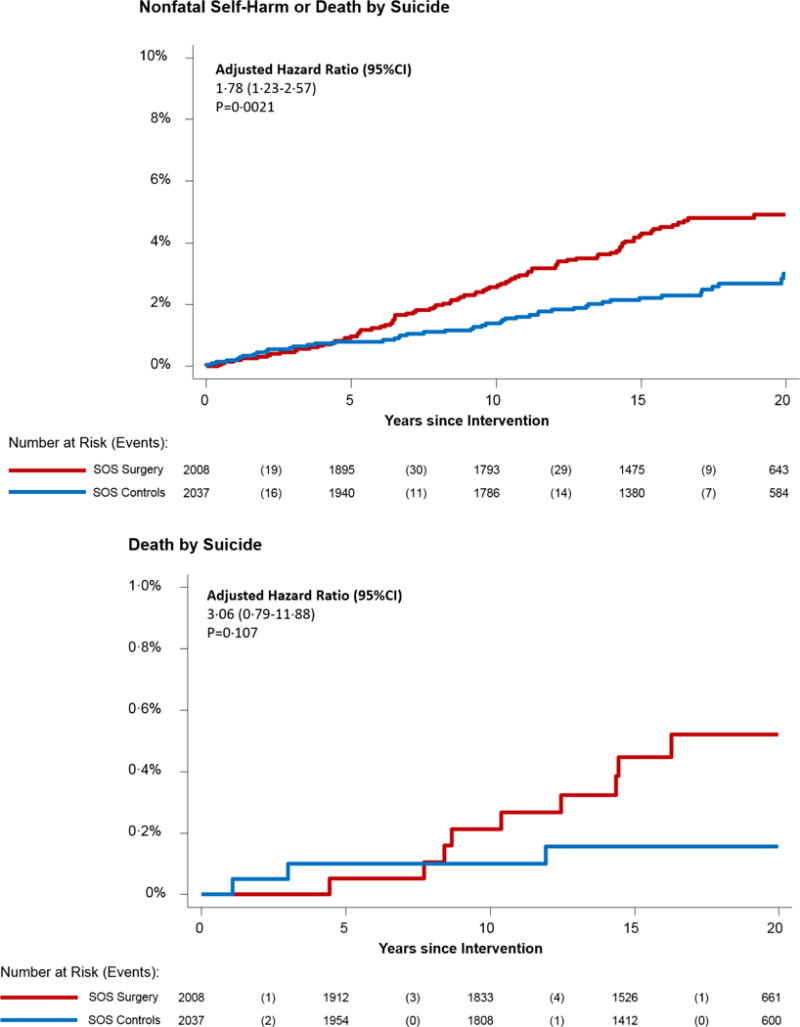
Cumulative incidence of suicide and nonfatal self-harm in the Swedish Obese Subjects (SOS) study Hazard ratios adjusted for age, sex, BMI, and history of self-harm

**Figure 2:**
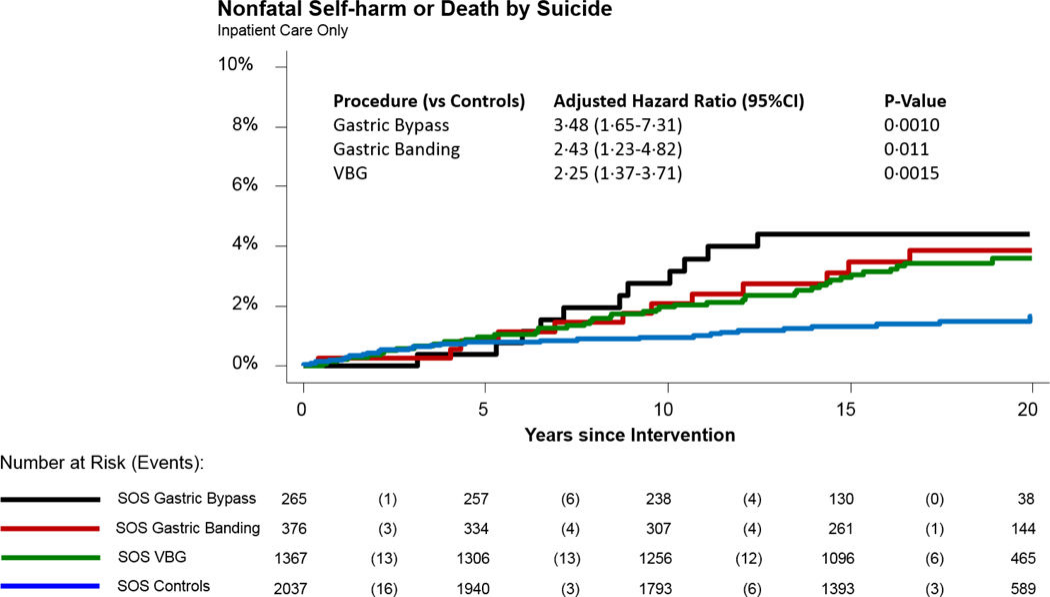
Cumulative incidence of suicide and nonfatal self-harm in the Swedish Obese Subjects (SOS) study by primary procedure type Case ascertainment from inpatient care and Causes of Death Register only as the outpatient care component was added in 2001 and gastric bypass was used more in the later part of the SOS recruitment period VBG=vertical-banded gastroplasty

**Figure 4:**
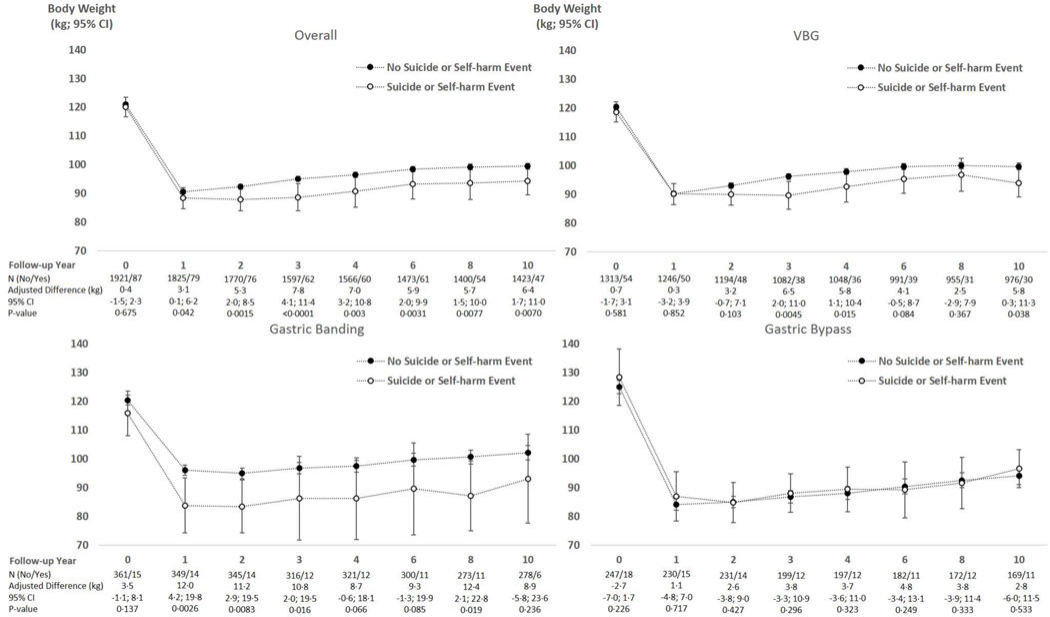
Weight development over 10 years in surgery patients in the SOS study by suicide and self-harm status (overall and by primary procedure type) Adjustment variables were the same as in the main analysis (age, sex, baseline BMI, and history of self-harm)

During 68,528 person-years (median 18; interquartile range 14–21) there were 87 versus 49 suicides or nonfatal self-harm events in the SOS surgery and control group, respectively (adjusted hazard ratio [aHR] 1.78 [95%CI 1.23–2.57]; P=0.0021), of which 9 and 3 were suicides (3.06 [0.79–11.88]; P=0.107; Figure 1). Additional adjustment for baseline diabetes and cardiovascular disease resulted in similar estimates for suicide or nonfatal self-harm (aHR 1.74 [1.20–2.52]; P=0.0033) and for suicide (3.33 [0.86–12.97]; P=0.083). In analyses by primary procedure type, increased risk of suicide or nonfatal self-harm was found for gastric bypass (aHR 3.48 *1.65–7.31+; P=0.0010), gastric banding (2.43 *1.23–4.82+; P=0.011) and vertical-banded gastroplasty (2.25 *1.37–3.71+; P=0.0015) versus controls (Figure 2, Figure 3A). Surgery patients who died by suicide or had a nonfatal self-harm event had similar or lower body weight during follow-up than patients who did not, while there was no difference at baseline (Figure 4).

Poisoning was the most common mode of suicide in SOS (78% [7/9] for surgery versus 100% [3/3] for controls; eTable3) and of nonfatal self-harm (70% [57/81] versus 53% [25/47]; eTable4). Out of 9 suicides in the surgery arm, 5 occurred in gastric bypass patients (2 who had primary gastric bypass, 2 who were converted from vertical-banded gastroplasty, 1 converted from gastric banding; eTable3). Substance abuse was recorded in 48% [39/81] of surgery patients and 28% [13/47] of controls with nonfatal self-harm events (P=0.023; eTable4).

**Figure 5:**
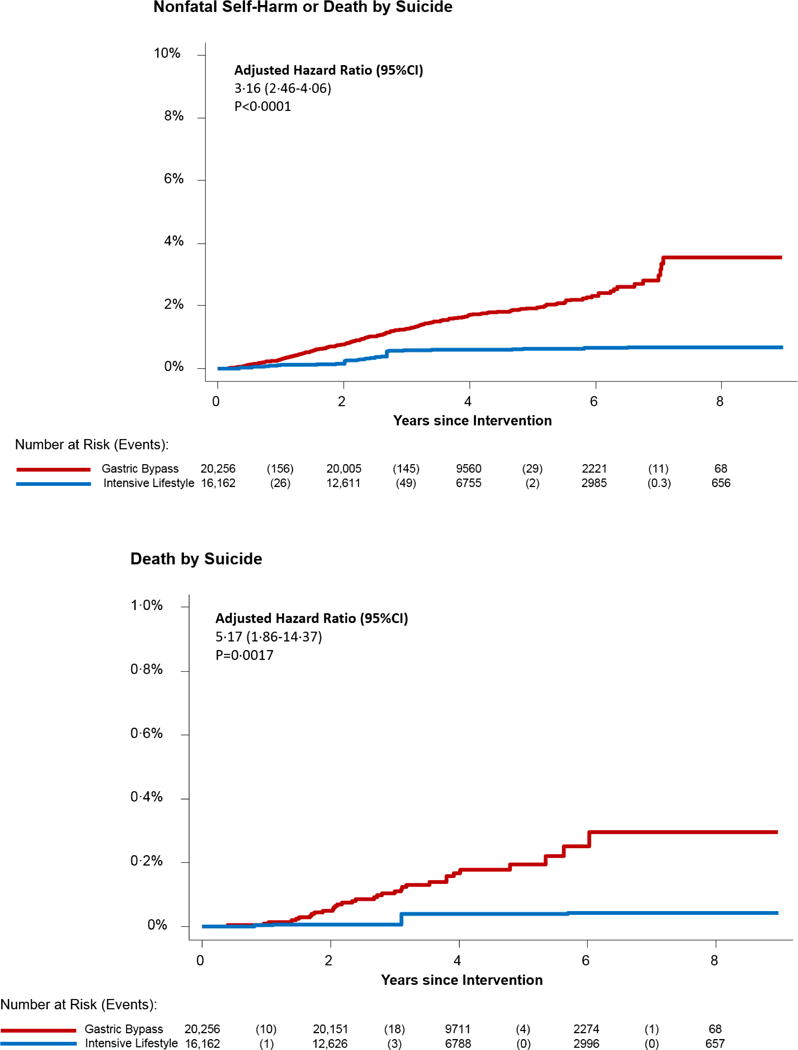
Cumulative incidence of suicide and nonfatal self-harm in the SOReg/Itrim study comparing gastric bypass with intensive lifestyle modification Matched on age, sex, BMI, education level, cardiovascular disease, diabetes, history of self-harm, substance abuse, visits in psychiatric care, use of antidepressants, and use of anxiolytics. Hazard ratios adjusted for age, BMI, income, marital status, disability pension, and unemployment N for intensive lifestyle group are weighted by the strata size to account for the matching

During 149,582 person-years (median 3.9; interquartile range 2.8–5.2) there were 341 suicides or nonfatal self-harm events in the SOReg gastric bypass group and 84 in the intensive lifestyle group (aHR 3.16 [2.46–4.06]; P<0.0001), of which 33 and 5 were suicides (5.17 [1.86–14.37]; P=0.0017; Figure 5). As in SOS, poisoning was the most common mode of suicide (79% [26/33] for surgery versus 80% [4/5] for intensive lifestyle; eTable3) and nonfatal self-harm (68% [214/316] versus 59% [47/80]; eTable4). Substance abuse diagnoses were more common after gastric bypass than intensive lifestyle in those with nonfatal self-harm events (51% [162/316] versus 29% [23/80], P=0.0003; eTable4).

In subgroup analyses, the risk of suicide or nonfatal self-harm was elevated in both SOS and SOReg/Itrim in surgery patients versus controls in the subgroup free of registered psychiatric disorders and without self-harm history at baseline (Figure 3). The risk was also elevated in both studies in the surgery group versus controls in those with as well as those without university education.

## DISCUSSION

We compared the risk of suicide and nonfatal self-harm after bariatric surgery and nonsurgical obesity treatment in two large matched cohorts, and in both of them, surgery patients were at an increased risk. However, despite certain psychiatric disorders being part of the exclusion criteria in the SOS study, surgery patients had almost twice the prevalence of self-harm history at baseline compared to controls (3.4% *69/2008+ versus 1.9% *38/2037+), and such a history is strongly related to future events.(12) Nevertheless, the increased risk was observed also in the subgroup of patients free of known psychiatric disorders and without self-harm history at baseline, in both SOS and SOReg/Itrim.

Strengths of this study include access to long-term information on self-harm, substance abuse and other psychiatric disorders in two large matched cohort studies of bariatric surgery and nonsurgically treated obese controls. Nationwide health registers enabled near complete outcome ascertainment for both suicide and nonfatal self-harm resulting in hospital care over up to 8 years in SOReg/Itrim and 27 years in SOS. Furthermore, the two cohorts complemented each other: SOS had very long follow-up, which by necessity means older surgical techniques than in SOReg/Itrim. SOS also had less intensive control treatment than SOReg/Itrim. Trials of bariatric surgery have been criticised for use of comparators of insufficient intensity, and very low calorie diets have been discussed as a component of higher intensity regimens.(22) In a meta-analysis of bariatric surgery trials, weight change during the first 2 years in controls ranged between +1kg to -8kg, while surgery patients lost a mean 20–43kg.(23) At one year in SOReg/Itrim, the intensive lifestyle modification resulted in a weight loss of 15% (18kg) compared to 32% (37kg) for gastric bypass.

A limitation of this study is that neither SOS nor SOReg/Itrim were randomised. However, it is unlikely that randomised trials of bariatric surgery will have sufficient power to investigate rare events such as suicide, necessitating observational designs. Both SOS and SOReg/Itrim included obese matched controls attempting to lose weight and accounted for multiple suicide risk factors, but selection bias and residual confounding may still have affected our results. The Itrim participants, in contrast to SOS controls, paid for weight loss treatment, while surgery patients were more likely to be referred and not pay out of pocket, which may be important as suicidal behavior displays a strong socioeconomic gradient. In subgroup analysis by education level, we observed elevated risk of suicide or nonfatal self-harm after surgery in both SOS and SOReg/Itrim in all education level strata. For SOReg/Itrim we also adjusted for income, disability pension, and unemployment to reduce bias from differences in socioeconomic position.

No patients in our cohorts had sleeve gastrectomy, a method which is increasingly used. Also, Swedes are predominantly Caucasian. We do not know if our results can be generalised to patients having sleeve gastrectomy or other ethnic groups. Regarding procedure type in SOS, the analyses were conducted according to primary procedure. This may overestimate the risks for vertical-banded gastroplasty and gastric banding, as conversion to gastric bypass was common. Regarding follow-up, due to the long recruitment in SOS and nationwide scope of SOReg/Itrim, it was not possible to provide detailed information on contacts with psychologists and primary care after treatment.

Finally, in contrast to SOReg/Itrim there was no between-group difference in suicide or nonfatal self-harm during the first 5 years in SOS. A potential explanation for this is that SOS is a prospective study with at least annual follow-up visits during the first 4 years, in addition to routine follow-up in the healthcare system. SOReg patients had less intensive follow-up. Furthermore, only 13% [265/2008] had gastric bypass in SOS compared with 100% in SOReg. In our analyses by procedure type in SOS, gastric bypass was associated with 3.4 times increased risk, gastric banding 2.4 and vertical-banded gastroplasty 2.3 versus controls.

Several mechanisms have been suggested for an increased risk of suicide after bariatric surgery,(11, 24) including disappointment among surgery patients due to insufficient weight loss, subsequent weight re-gain, recurrence of obesity-related comorbidities after initial remission, or that weight loss did not have the expected life-changing effects.(11) However, rather than insufficient weight loss, we found that SOS surgery patients who later died by suicide or had a nonfatal self-harm event had either similar or greater weight loss than those who did not, irrespective of primary procedure type.

Previous studies, including SOS, have shown increased incidence of alcohol and substance abuse after gastric bypass,(9) and this could result in impulsive acts. Also, a certain alcohol intake has been reported to result in higher blood alcohol concentrations after compared to before gastric bypass.(25) The effect on uptake of other substances is largely unknown but it is possible that gastric bypass patients more easily get intoxicated. The higher incidence of alcohol abuse after gastric bypass compared to restrictive procedures(26–28) may partly explain why the hazard ratios for suicide and nonfatal self-harm are higher in SOReg/Itrim (gastric bypass only) than in SOS (13% gastric bypass [265/2008]). In SOS, the risk of alcohol abuse diagnosis was 5 times higher after gastric bypass and twice as high after vertical-banded gastroplasty compared to controls, while no difference was detected after gastric banding.(27)

Mental health problems are much more prevalent in patients undergoing bariatric surgery than in age-sex-matched general population comparators.(13) The 4-year trajectory of antidepressant use after surgery has been reported to be similar to that in the general population, while steeper for benzodiazepines, hypnotics and sedatives.(13) The association between bariatric surgery, different procedure types, and mental health is not yet well-described based on randomised trials or carefully matched cohort studies with obese control groups attempting to lose weight.(10) In SOS, no difference between surgery and controls in overall psychiatric drug use has been found.(29) For SOReg/Itrim, a higher incidence of hypnotic/sedative use and higher dose increases in prevalent users have recently been reported after gastric bypass versus intensive lifestyle modification.(30)

Other proposed mechanisms behind an association between bariatric surgery and suicide include neuroendocrine alterations, exacerbations of depression and anxiety due to micro-/macro-nutrient deficiencies caused by malabsorption, and psychological mechanisms like maladaptive eating behaviors.(31) In SOS, health-related quality of life has been shown to be improved up to 10 years after surgery compared to baseline, and also higher compared to the control group.(32) However, average improvements may mask deteriorating quality of life in a subset of patients due to, for example, surgical complications or alcohol abuse. For a rare event such as suicide, such a subset does not need to be large to produce statistically significant risk increases.

Our observational findings indicate a need for patient information before surgery regarding self-harm and post-operative psychiatric surveillance, as recently suggested.(33) However, it may be difficult to design such a surveillance system, given the rarity of suicides: we observed 42 suicides in SOS and SOReg over 117,000 person-years after surgery. Hence annual psychiatric surveillance is likely to be inefficient. Restricting surveillance to high risk patients, for example those with baseline psychiatric disorders, would be more efficient but applied to our data this strategy would miss almost 50% dying by suicide.

Current international guidelines list active or recent substance abuse as a contraindication to surgery.(34) Psychiatric hospitalisation and self-harm history are considered risk factors for poor outcomes but not a contraindication when appropriate mental health treatment is provided. Further, the European guidelines recommend pre-operative psychological assessment by a psychiatrist or psychologist not just for diagnostic purposes but also to identify areas of vulnerability, and higher-risk patients should be selected for post-operative monitoring.(35)

Despite our finding of an increased risk of suicide we do not believe that our findings at this point should discourage use of bariatric surgery, at least not from a survival perspective. Several well-designed observational studies show a survival benefit versus obese controls despite a potential increased suicide risk.(2–4) While the relative risk of suicide is high, the absolute risk is low. For example, in the Utah Mortality Study the incidence of all-cause mortality in surgery and matched control participants was 37.6 and 57.1 per 10,000 person-years, respectively, compared to 2.6 and 0.9 for suicide.(2) Beyond mortality, the many documented and common benefits of bariatric surgery(9, 10) are likely to outweigh our finding of an increased risk of suicide and self-harm, but our observations could help to inform and refine guidelines regarding how surgery candidates are selected and followed over time.

In conclusion, we found a positive association between bariatric surgery and suicide or nonfatal self-harm. We also found that history of self-harm was more common in patients choosing surgery than in individuals choosing nonsurgical treatment.

## Supplementary Material



## Figures and Tables

**Figure 3A:**
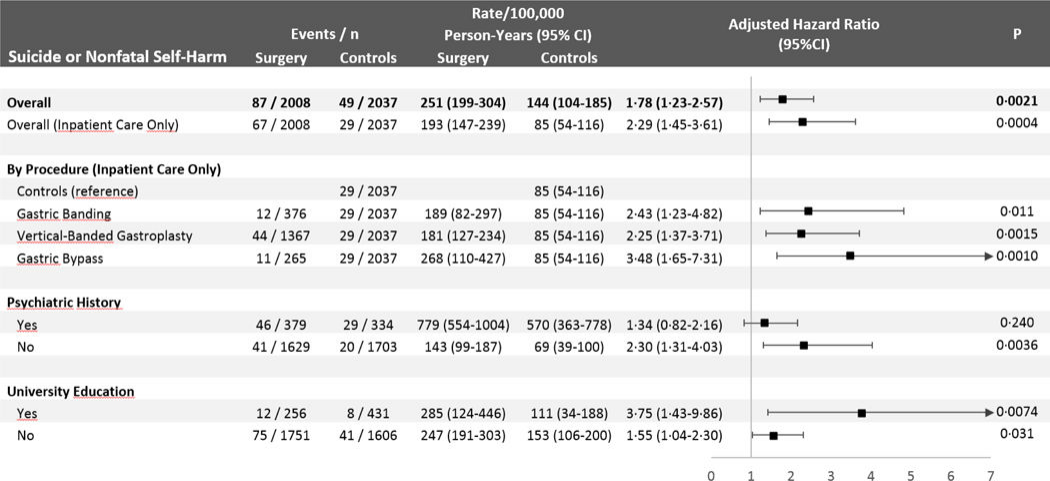
Suicide and nonfatal self-harm in the Swedish Obese Subjects (SOS) cohort overall and by subgroups Adjusted for age, sex, BMI, and history of self-harm. Inpatient care only: Refers to case ascertainment excluding data from the outpatient component from the National Patient Register. Outpatient data were available from 2001 and onwards. Psychiatric history: Baseline characteristics for the subgroup with psychiatric history are provided in eTable5.

**Figure 3B:**
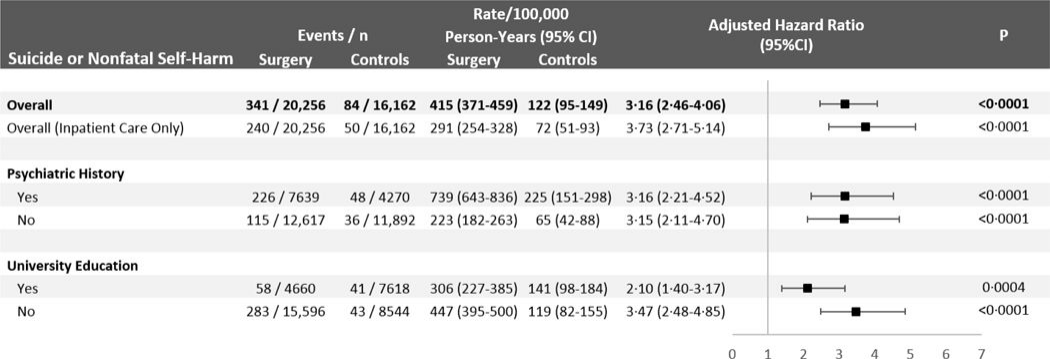
Suicide and nonfatal self-harm in the SOReg/Itrim cohort overall and by subgroups Matched on age, sex, BMI, education level, cardiovascular disease, diabetes, history of self-harm, substance abuse, visits in psychiatric care, use of antidepressants, and use of anxiolytics. Additional adjustment was made for age, BMI and income as continuous variables, as well as for marital status, disability pension, and unemployment status as binary variables. Incidence rates and hazard ratios are weighted by the strata size to account for the matching. Inpatient care only: Refers to case ascertainment excluding data from the outpatient component from the National Patient Register. Psychiatric history: Baseline characteristics for the subgroup with psychiatric history are provided in eTable5.

**Table 1:** Participant characteristics at baseline

	Swedish Obese Subjects Recruitment: 1987–2001			SOReg/Itrim Recruitment: 2006–2013		
	Bariatric Surgery^a^ (n=2008)	Controls (n=2037)	P	Gastric Bypass (n=20,256)	Intensive Lifestyle (16,162)	P
Women, n (%)	1420 (70.7%)	1447 (71.0%)	0.824	16,071 (79.3%)	12,823 (79.3%)	1.0
Age (Years), Mean (SD)	47.2 (5.9)	48.7 (6.3)	<0.0001	41.3 (10.5)	41.5 (10.8)	0.125
Body-Mass Index (kg/m^2^), Mean (SD)	42.4 (4.5)	40.1 (4.7)	<0.0001	41.1 (3.9)	40.6 (4.1)	<0.0001
University Education, n (%)	256 (12.7%)	431 (21.2%)	<0.0001	4660 (23.0%)	3718 (23.0%)	1.0
Married, n (%)	–	–	–	9034 (44.6%)	6837 (42.3%)	<0.0001
Income (1000 €), Mean (SD)	–	–	–	23.7 (14.5)	28.2 (19.4)	<0.0001
Disability Pension, n (%)	357 (17.8%)	316 (15.5%)	0.053	2366 (11.7%)	997 (6.2%)	<0.0001
Unemployment, n (%)	–	–	–	2016 (10.0%)	883 (5.5%)	<0.0001
**History of Psychiatric Illness, n (%)**						
Self-Harm	69 (3.4%)	38 (1.9%)	0.0019	403 (2.0%)	322 (2.0%)	1.0
Substance Abuse	58 (2.9%)	49 (2.4%)	0.339	294 (1.5%)	235 (1.5%)	1.0
Psychiatric healthcare visits^b^	200 (10.0%)	175 (8.6%)	0.133	3083 (15.2%)	2460 (15.2%)	1.0
Use of Antidepressants	133 (6.6%)	114 (5.6%)	0.173	6108 (30.2%)	4873 (30.2%)	1.0
Use of Anxiolytics	98 (4.9%)	88 (4.3%)	0.395	3446 (17.0%)	2750 (17.0%)	1.0
Use of Hypnotics and Sedatives	74 (3.7%)	59 (2.9%)	0.160	4426 (21.9%)	2888 (17.9%)	<0.0001
**Physical Health Status, n (%)**						
Diabetes	346 (17.2%)	263 (12.9%)	0.0001	1954 (9.6%)	1559 (9.6%)	1.0
Cardiovascular Disease	383 (19.1%)	260 (12.8%)	<0.0001	4203 (20.7%)	3354 (20.7%)	1.0

a Primary operations were: 1367 (68.1%) vertical-banded gastroplasty, 376 (18.7%) gastric banding, 265 (13.2%) gastric bypass

b SOS: Only from inpatient care; SOReg: From both inpatient (6.7% surgery versus 6.5% intensive lifestyle, P=0.439) and hospital-based outpatient care (12.4% surgery versus 12.3% intensive lifestyle, P=0.745)

## RESEARCH IN CONTEXT

### Evidence before this study

A recent systematic review for the US National Institutes of Health concluded that emerging data indicate an increased risk of suicide, or deaths not caused by disease, after bariatric surgery. The cited observational studies used comparators with obesity who applied for driver’s licenses or were seeking but did not receive bariatric surgery. There are no reports on the risk of suicide after bariatric surgery versus nonsurgical weight loss therapy. Further, previous studies have not accounted for baseline differences in psychiatric status such as history of self-harm, substance abuse and depression.

### Added value of this study

Based on two large long-term matched controlled studies of individuals with obesity intending to lose weight, we found a substantially increased relative risk of suicide or nonfatal self-harm in the surgery group, after accounting for baseline psychiatric status.

The excess risk after surgery was not explained by insufficient weight loss or weight regain, as individuals dying by suicide or who had hospital treatment for nonfatal self-harm had similar or greater weight loss during follow-up than other patients.

Despite our attempts to match and stratify the analyses by baseline history of self-harm, substance abuse, depression and anxiety, we cannot rule out in our nonrandomised studies that the observed increased risk of suicide or nonfatal self-harm after bariatric surgery is simply due to different patient characteristics among individuals who chose surgery instead of nonsurgical weight loss methods.

### Implications of all the available evidence

It is unlikely that there will ever be a randomised trial large and long enough to assess the risk of suicide between bariatric surgery and a nonsurgical intervention. Our matched cohort studies and previous observational studies indicate that bariatric surgery is associated with an increased risk of suicide. The absolute suicide risk is small and the association may be influenced by selection bias and residual confounding. However, the relative risk of suicide and nonfatal self-harm is considerable even when accounting for multiple known suicide risk factors. As bariatric surgery, and especially gastric bypass, is associated with increased risk of alcohol and substance abuse, there is also a plausible mechanism behind an increased suicide risk.

## ABBREVIATIONS

BMI: body mass index (kg/m^2^)
LCD/VLCD: low calorie diet/very low calorie diet
SOReg: Scandinavian Obesity Surgery Registry
SOS: study Swedish Obese Subjects study
