# Supplementary material for: BARIATRIC SURGERY & SUICIDE: RESULTS FROM TWO CONTROLLED MATCHED COHORT STUDIES
Source: Lancet Diabetes Endocrinol. Author manuscript; Available in PMC 2019 Mar 1. (PMC5932484; doi:10.1016/S2213-8587(17)30437-0)
Supplement: Supplementary file 1 [file NIHMS935572-supplement-3.pdf]

Table of Contents

Item	Description
eMethods	Very low / low calorie diets (VLCD/LCD) in the intensive lifestyle program
	Indications & follow-up after bariatric surgery in Sweden
eTable 1	Psychosocial variables and personality traits used in the matching algorithm
eTable 2	International Classification of Diseases (ICD) codes and Anatomical Therapeutic Chemical (ATC) classification system codes used
eTable 3	Description of deaths by suicide
eTable 4	Description of nonfatal self-harm events (excluding individuals dying by suicide who did not have a nonfatal self-harm event after intervention)
eTable 5	Baseline characteristics in participants with history of psychiatric disorder or nonfatal self-harm
eTable 6	Evidence table of studies on bariatric surgery and suicide
eTable 7	Distribution by BMI category in the SOReg/Itrim study
eFigure 1	Flow chart for the Swedish Obese Subjects (SOS) study
eFigure 2	Flow chart for the SOReg/Itrim study of gastric bypass and low/very low calorie diet treated individuals (intervention years: 2006-2013)
References	

eMethods

Very Low / Low Calorie Diets (VLCD/LCD) in the Intensive Lifestyle Program

- VLCD: Liquid-based formula diet of 500kcal/day for 3-10 weeks (125kcal/sachet, 4 sachets/day, approved as sole source VLCD by the Swedish National Food Agency) followed by 2-8 weeks gradual introduction of normal food. Early introduction of normal food occurred if patients were satisfied with the achieved weight loss or reached BMI<25kg/m^2. In June, 2009, the program was changed to 600 kcal/day for 3 weeks followed by 800kcal/day for up to 9 weeks.

- LCD: 2 calorie-restricted normal food meals and 2 formula-diet meal replacement sachets per day providing a total caloric intake of about 1200-1500kcal.

Indications & Follow-Up After Bariatric Surgery in Sweden

In the SOS study, patients underwent surgery between 1987 and 2001 at 25 surgical departments. During this time there were no uniform national guidelines for follow-up after bariatric surgery. Most follow-up visits were done by the bariatric surgeons and were focused on surgical and medical complications as well as nutritional and gastrointestinal problems. However, Sweden has a tax funded universally accessible health care system including access to psychologists and psychiatrists as well as internists, surgeons, primary care physicians, and dietitians. There has never been general use of psychological behavioural treatment or counselling before or after bariatric surgery in Sweden but if needed this was solved on an individual basis.

With growing numbers of operations in Sweden, specialist nurses were trained to handle much of the follow-up, and when SOReg started in 2007 this system for follow-up was fully developed. Certified nurses in Sweden have an academic education and work more independently than nurses in many other European countries and the US. If physical or psychological problems are identified patients can be referred to psychologists, psychiatrists, surgeons and dietitians associated with the surgical department. SOReg has implemented follow-up visits at 6 weeks (95% of patients covered), 1 year (87% covered), 2 years (65% covered), and recently also follow-up at 5 and 10 years.

The general recommendations regarding preoperative assessment include a psychiatric evaluation in individuals with psychiatric problems or eating disorders. Psychiatric disorders are relative contraindications and patients with such disorders may be accepted for surgery after psychiatric evaluation. Candidates for surgery are also required to have a stable psychosocial situation. Ongoing alcohol or substance abuse constitute absolute contraindications. Previous alcohol or substance abuse without documented abstinence for at least 2 years also constitute absolute contraindications. Previous alcohol or substance abuse more than 2 years back constitute relative contraindications.

eTable 1 Psychosocial variables and personality traits used in the matching algorithm

	Swedish Obese Subjects Recruitment: 1987-2001		
Mean (SD)	Bariatric Surgery (n=2010)	Controls (n=2037)	P
Current Health Score (9-36, higher better)	21·4 (6·1)	22·7 (6·2)	<0·001
Monotony Avoidance Score[a] (10-40)	22·5 (5·1)	22·6 (5·0)	0·525
Psychasthenia Score[b] (10-40)	23·9 (5·2)	23·2 (5·3)	<0·001
Quantity of Social Support (0-12)	6·0 (2·4)	6·1 (2·5)	0·483
Quality of Social Support (0-5)	4·3 (1·3)	4·3 (1·3)	0·551
Stressful Life Events (0-8)	2·5 (1·3)	2·4 (1·3)	0·091

eTable 2 International Classification of Diseases (ICD) codes and Anatomical Therapeutic Chemical (ATC) classification system codes used

Condition	ICD-10	ICD-9	ICD-8	ATC Code
Substance Abuse	F10-19	291-292, 303-305	291; 294,3; 303-304	N07BB, N07BC
Attempted Suicide/Self-Harm	X60-84 Y10-34, Y870	E950-959 E980-989	E950-959 E980-989	
Suicide – Undetermined Intent	Y10-34, Y870	E980-989	E980-989	
Suicide - Poisoning	X60-X69 Y10-Y19	E950-E952, E980-E982	E950-E952, E980-E982	
Psychiatric Care or Any Psychiatric Drug	Chapter F	290-319	290-315	N05, N06
Antidepressants				N06A
Antidiabetic Drugs				A10
Anxiolytic Drugs				N05B
Hypnotic or Sedative Drugs				N05C
Cardiovascular Disease	Chapter I	390-459	390-458	

Events of undetermined intent are usually included in Swedish suicide research. Several studies have reported a large fraction of definite suicides when re-evaluating such events.(1, 2)

[a] Monotony avoidance is a personality trait characterised by abnormal attempts to avoid routine and to seek change and action (i.e., thrill- or sensation-seeking behaviour)
[b] Psychasthenia is characterised by tiredness, concentration and memory difficulties, and various sensations including palpitations

eTable 3 Description of deaths by suicide

	Swedish Obese Subjects Recruitment: 1987-2001		SOReg/Itrim Recruitment: 2006-2013	
	**Bariatric Surgery (n=9)**	**Controls (n=3)**	**Gastric Bypass (n=33)**	**Intensive Lifestyle (n=5)**
**Suicide**	**9 (100%)**	**3 (100%)**	**33 (100%)**	**5 (100%)**
- Undetermined Intent	2 (22%)	1 (33%)	10 (30%)	3 (60%)
**Mode of Suicide**				
- Poisoning	7 (78%)	3 (100%)	26 (79%)	4 (80%)
- Other	2 (22%)	0 (0%)	7 (21%)	1 (20%)
**Non-Fatal Self-Harm Event**				
- Before Baseline	2 (22%)	0 (0%)	7 (21%)	1 (20%)
- After Intervention	3 (33%)	1 (33%)	8 (24%)	1 (20%)
**Previous Substance Abuse**				
- Before Baseline	2 (22%)	0 (0%)	5 (15%)	2 (40%)
- After Intervention	2 (22%)	0 (0%)	10 (30%)	1 (20%)
**Season**[c]				
Spring (March-May)	1 (11%)	0 (0%)	7 (21%)	0 (0%)
Summer (June-August)	1 (11%)	0 (0%)	7 (21%)	3 (60%)
Fall (September-November)	3 (33%)	0 (0%)	12 (36%)	1 (20%)
Winter (December-February)	4 (44%)	3 (100%)	7 (21%)	1 (20%)
**Primary Bariatric Procedure**				
Gastric Bypass	2 (22%)	-	33 (100%)	-
Gastric Banding	2 (22%)	-	-	-
VBG[d]	5 (56%)	-	-	-
**Revision Surgery**				
VBG to Gastric Bypass	2 (22%)	-	-	-
Banding to Gastric Bypass	1 (11%)	-	-	-

[c] Calendar definition (source: Swedish Meteorological and Hydrological Institute [SMHI]); https://www.smhi.se/kunskapsbanken/meteorologi/arstider-1.1082)
[d] Vertical-banded gastroplasty

eTable 4 Description of nonfatal self-harm events (excluding individuals dying by suicide who did not have a nonfatal self-harm event after intervention)

	Swedish Obese Subjects Recruitment: 1987-2001		SOReg/Itrim Recruitment: 2006-2013	
	**Bariatric Surgery (n=81)**	**Controls (n=47)**	**Gastric Bypass (n=316)**	**Intensive Lifestyle (n=80)**
**Nonfatal Self-Harm Events**	**81 (100%)**	**47 (100%)**	**316 (100%)**	**80 (100%)**
- Undetermined Intent	32 (40%)	20 (43%)	85 (27%)	33 (41%)
**Mode of Self-Harm**				
- Poisoning	57 (70%)	25 (53%)	214 (68%)	47 (59%)
- Other	24 (30%)	22 (47%)	102 (32%)	33 (41%)
**Baseline Nonfatal Self-Harm**	17 (21%)	4 (9%)	41 (13%)	13 (16%)
**Substance Abuse**				
- Before Baseline	11 (14%)	3 (6%)	35 (11%)	13 (16%)
- After Intervention	39 (48%)	13 (28%)	162 (51%)	23 (29%)
**Season**[e]				
Spring (March-May)	21 (26%)	13 (28%)	64 (20%)	19 (24%)
Summer (June-August)	16 (20%)	11 (23%)	80 (25%)	18 (23%)
Fall (September-November)	17 (21%)	14 (30%)	93 (29%)	24 (30%)
Winter (December-February)	27 (33%)	9 (19%)	79 (25%)	19 (24%)
**Primary Bariatric Procedure**				
Gastric Bypass	16 (20%)	-	316 (100%)	-
Gastric Banding	14 (17%)	-	-	-
VBG[f]	51 (63%)	-	-	-
**Revision Surgery**				
VBG to Gastric Bypass	10 (12%)	-		
Banding to Gastric Bypass	3 (4%)	-		

[e] Calendar definition (source: Swedish Meteorological and Hydrological Institute [SMHI]); https://www.smhi.se/kunskapsbanken/meteorologi/arstider-1.1082)

[f] Vertical-banded gastroplasty

eTable 5 Baseline characteristics in participants with history of psychiatric disorder or nonfatal self-harm

	**Swedish Obese Subjects** Recruitment: 1987-2001			**SOReg/Itrim** Recruitment: 2006-2013		
	**Bariatric Surgery (n=379)**	**Controls (n=334)**	**P**	**Gastric Bypass (n=7639)**	**Intensive Lifestyle (n=4270)**	**P**
Women, n (%)	289 (76·3%)	240 (71·9%)	0·181	6917 (90·5%)	3866 (90·5%)	1·0
Age (Years), Mean (SD)	48·1 (6·0)	49·3 (6·5)	0·0092	42·0 (10·0)	42·2 (10·3)	0·501
Body Mass Index (kg/m^2), Mean (SD)	42·0 (4·4)	40·1 (4·8)	<0·0001	40·4 (3·7)	39·8 (3·9)	<0·0001
University Education, n (%)	44 (11·6%)	56 (16·8%)	0·048	1795 (23·5%)	1003 (23·5%)	1·0
Married, n (%)	-	-	-	3348 (43·8%)	1777 (41·6%)	0·019
Income (1000 €), Mean (SD)	-	-	-	21·7 (12·1)	26·2 (15·5)	<0·0001
Disability Pension, n (%)	-	-	-	1617 (21·2%)	525 (12·3%)	<0·0001
Unemployment, n (%)	-	-	-	769 (10·1%)	314 (7·4%)	<0·0001
**History of Psychiatric Illness, n (%)**						
Self-Harm	69 (18·2%)	38 (11·4%)	0·011	403 (5·3%)	225 (5·3%)	1·0
Substance Abuse	58 (15·3%)	49 (14·7%)	0·813	294 (3·8%)	164 (3·8%)	1·0
Psychiatric Hospital Visits[7]	200 (52·8%)	175 (52·4%)	0·920	3083 (40·4%)	1723 (40·4%)	1·0
Use of Antidepressants	133 (35·1%)	114 (34·1%)	0·788	6108 (80·0%)	3414 (80·0%)	1·0
Use of Anxiolytics	98 (25·9%)	88 (26·3%)	0·882	3446 (45·1%)	1926 (45·1%)	1·0
Use of Hypnotics & Sedatives	74 (19·5%)	59 (17·7%)	0·525	3316 (43·4%)	1632 (38·2%)	<0·0001
**Physical Health Status, n (%)**						
Diabetes	76 (20·1%)	49 (14·7%)	0·059	434 (5·7%)	243 (5·7%)	1·0
Cardiovascular Disease	90 (23·7%)	59 (17·7%)	0·046	1521 (19·9%)	850 (19·9%)	1·0

[7] SOS: Inpatient care only; SOReg/Itrim: Inpatient care (17·8% surgery versus 17·2% intensive lifestyle; P=0·460) and outpatient care (32·8% versus 32·5%; P=0·739)

eTable 6 Evidence table of studies on bariatric surgery and suicide

Author<br>Journal, Year<br>Country	Intervention<br>Intervention years<br>Follow-up	Comparator	Suicide/self-harm
**Vs Obese Comparators**			
**Adams et al** (3)<br>NEJM, 2007<br>USA	Gastric bypass (n=9949)<br>1984-2002<br>Mean follow-up 7·1y	Severely obese individuals applying for driver's licenses (n=7925)	15 vs 5 suicides (2·6 vs 0·9 per 10,000 pyrs)<br>Adjusted HR: 2·03 (95%CI 0·66-6·27), P=0·22
**Adams et al** (4)<br>JAMA, 2012<br>USA	Gastric bypass (n=418)<br>2000-2011<br>Median follow-up: 5·8y	Severely obese individuals seeking but not receiving surgery (n=417)	4 vs 0 suicides (difference not statistically significant)
		Severely obese individuals drawn at random from the Utah Health Family Tree Program database (n=321)	4 vs 0 suicides (difference not statistically significant)
		Both control groups combined	4 vs 0 suicides<br>Odds ratio 18 (95%CI 1-385), P=0·02
**Kovacz et al** (5)<br>Acta Psych, 2017<br>Denmark	KJDF00-KJDF98 (n=9641)[8]<br>1997-2013<br>Mean follow-up: 4·0y	Individuals with a hospital diagnosis of obesity (n=7973)	HR for suicide (adjusted for sex and age):<br>1·35 (95%CI 0·36-5·07), P=0·658<br>HR for intentional self-harm:<br>3·23 (95%CI 1·93-5·40), P<0·001
**Vs General Population**			
**Backman et al** (6)<br>BJS, 2016<br>Sweden	Gastric bypass (n=16,755)<br>2001-2010<br>Follow-up: 4y	Age-sex-matched general population (n=167,550)	21·2 vs 10·6 suicide attempts per 10,000 pyrs<br>Adjusted HR: 2·85 (95%CI 2·40-3·39)
**Trolle-Lagerros et al** (7)<br>Ann Surg, 2017<br>Sweden	Gastric bypass (n=22,539)<br>2008-2012<br>Follow-up: 2y	General population via estimation of standardised mortality ratios (SMRs)	13/4 suicides in women/men<br>SMR women: 4·50 (95%CI 2·50-7·50)<br>SMR men: 1·71 (95%CI 0·54-4·12)
**Tindle et al** (8)<br>Am J Med, 2010<br>USA	Bariatric surgery (n=16,683)<br>1995-2004<br>Follow-up: Until December 2006	General population (35-64y)	31 suicides after bariatric surgery<br>Incidence per 10,000pyrs (women/men):<br>Bariatric surgery: 13·7 / 5·2<br>United States general population: 2·4 / 5·7
**Morgan & Ho (9)**<br>Ann Surg, 2017<br>Australia	Gastric banding, gastric bypass, sleeve gastrectomies, ileo-pancreatic diversions (n=12,062)<br>2007-2011<br>Follow-up: 40·6 months	General population	Higher rate of hospitalisations due to deliberate self-harm for bariatric surgery patients than the general population:<br>IRR 1·47 (95%CI 1·11–1·94), P=0·005
**Gribsholt et al (10)**<br>SOARD, 2017<br>Denmark	Gastric bypass (n=9895)<br>2006-2010<br>Follow-up: Until end of 2013	Age-sex-matched general population (n=247,366)	Mortality rate ratio for suicide:<br>2·78 (95%CI 1·44-5·33)

[8] Individuals with psychiatric contacts before surgery were excluded from the analysis of suicide after surgery (surgery group: 9487 women + 3125 men - 2971 with psychiatric contact; comparator group: 8598 women + 1241 men – 1866 with psychiatric contact)

eTable 6 Evidence table of studies on bariatric surgery and suicide (continued)

Author<br>Journal, Year<br>Country	Intervention<br>Intervention years<br>Follow-up	Comparator	Suicide/self-harm
**Before/After**			
**Bhatti et al** (11)<br>JAMA Surg, 2015<br>Canada	Gastric bypass (n=8681), intestinal bypass (n=89), sleeve gastrectomy (n=45)<br>2006-2011<br>Follow-up: 3y	-	Self-harm emergencies (per 1000pyrs)<br>2·33 before vs 3·63 after surgery<br>Rate ratio 1·54 (95%CI 1·03-2·30), P=0·007
**Kovacz et al** (5)<br>Acta Psych Scand, 2017<br>Denmark	KJDF00-KJDF98 (n=12,612)<br>1997-2013<br>Follow-up: 3, 5 and 7y	-	Intentional self-harm HRs:<br>1y: 1·71 (95%CI 1·14-2·55), P<0·01<br>3y: 1·60 (95%CI 1·09-2·33), P<0·05<br>5y: 1·91 (95%CI 1·24-2·93), P<0·01
**Morgan & Ho** (9)<br>Ann Surg, 2017<br>Australia	Gastric banding, gastric bypass, sleeve gastrectomies, ileo-pancreatic diversions (n=12,062)<br>2007-2011<br>Follow-up: 40·6 months	-	No statistically significant increase in deliberate self-harm hospitalisations after vs before surgery: IRR 0·79 (95%CI 0·54–1·16), P=0·21)

eTable 7 Distribution by BMI category in the SOReg/Itrim study

	SOReg/Itrim Recruitment: 2006-2013	
**BMI Category (kg/m^2)**	**Gastric Bypass (n=20 256)**	**Intensive Lifestyle (n=16 162)**
30 to <35	888 (4.4%)	709 (4.4%)
35 to <40	7519 (37.1%)	5999 (37.1%)
40 to <45	8255 (40.8%)	6587 (40.8%)
45 to <50	3594 (17.7%)	2868 (17.7%)

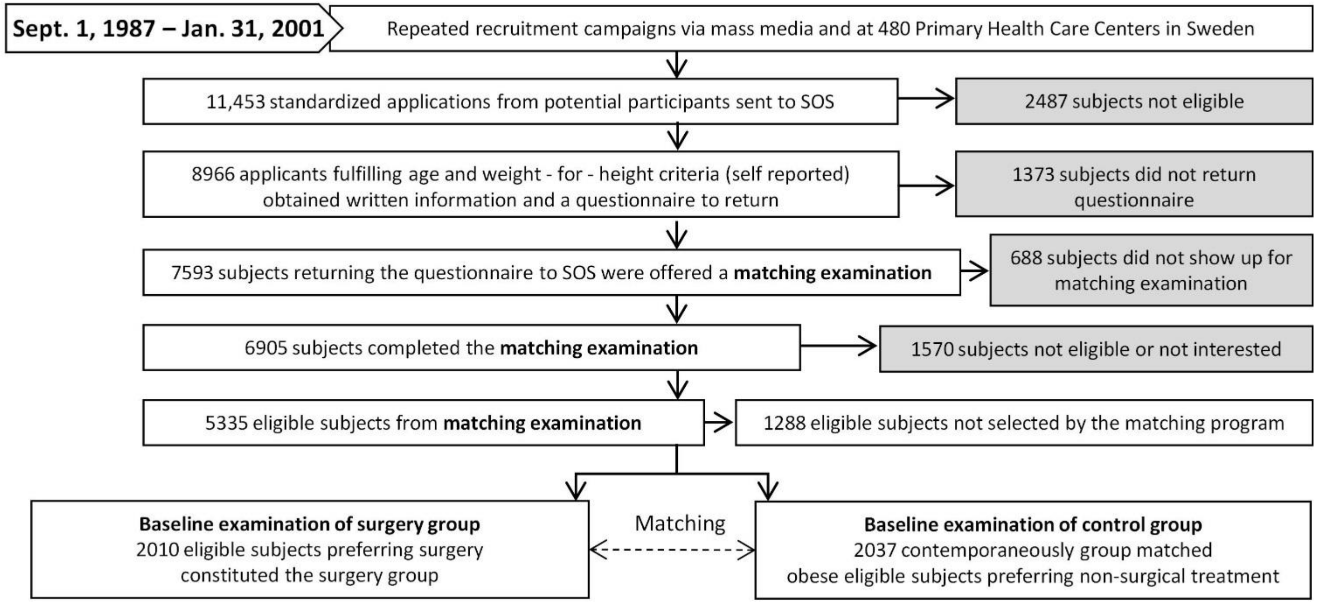

eFigure 1 Flow chart for the Swedish Obese Subjects (SOS) study

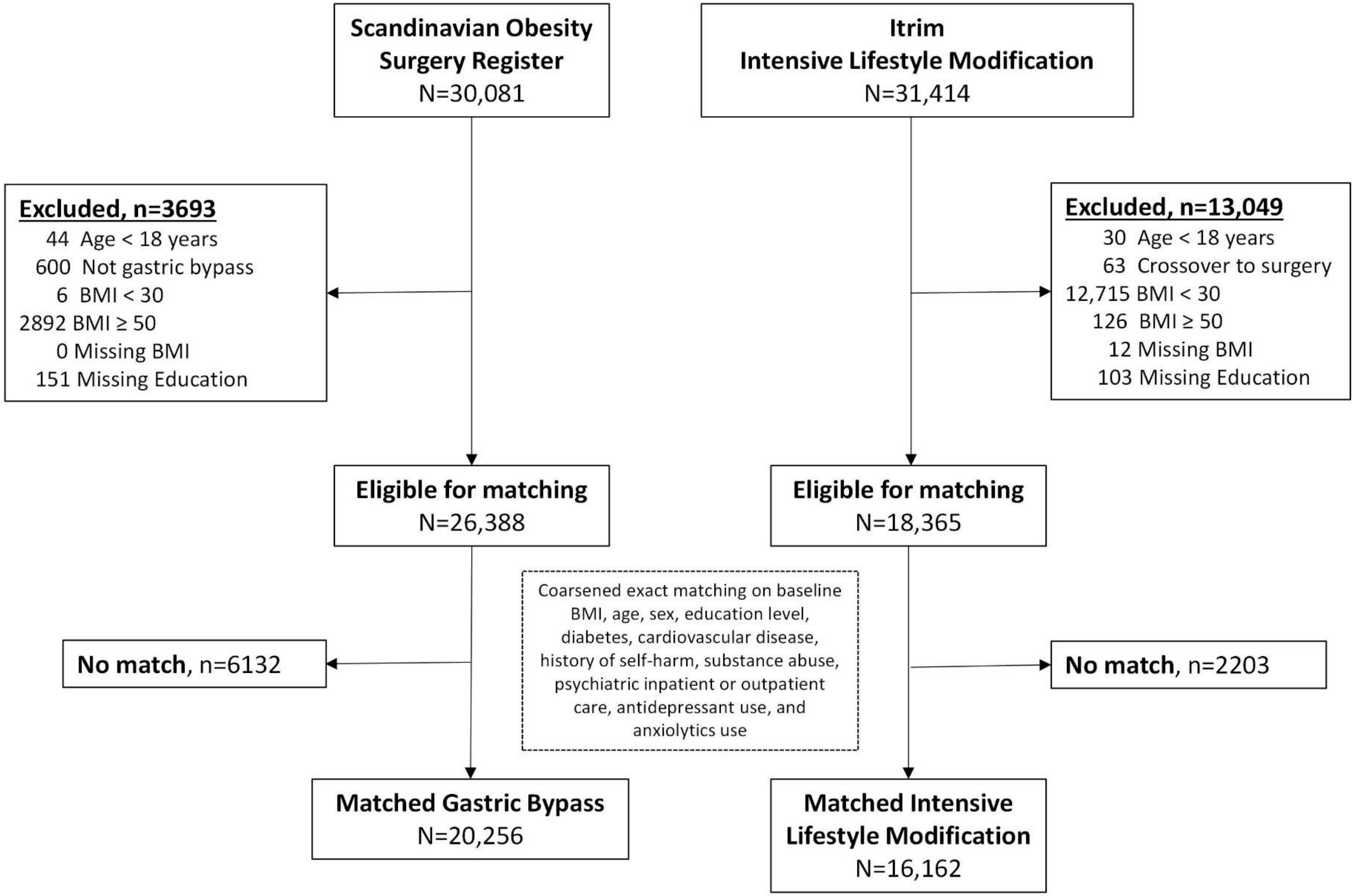

eFigure 2 Flow chart for the SOReg/Itrim study of gastric bypass and low/very low calorie diet treated individuals (intervention years: 2006-2013)

REFERENCES

1. Allebeck P, Allgulander C, Henningsohn L, Jakobsson SW. Causes of death in a cohort of 50,465 young men--validity of recorded suicide as underlying cause of death. Scand J Soc Med. 1991;19(4):242-7.

2. Ohberg A, Lonnqvist J. Suicides hidden among undetermined deaths. Acta Psychiatr Scand. 1998;98(3):214-8.

3. Adams TD, Gress RE, Smith SC, et al. Long-term mortality after gastric bypass surgery. N Engl J Med. 2007;357(8):753-61.

4. Adams TD, Davidson LE, Litwin SE, et al. Health benefits of gastric bypass surgery after 6 years. JAMA. 2012;308(11):1122-31.

5. Kovacs Z, Valentin JB, Nielsen RE. Risk of psychiatric disorders, self-harm behaviour and service use associated with bariatric surgery. Acta Psychiatr Scand. 2017;135(2):149-58.

6. Backman O, Stockeld D, Rasmussen F, Naslund E, Marsk R. Alcohol and substance abuse, depression and suicide attempts after Roux-en-Y gastric bypass surgery. The British Journal of Surgery. 2016;103(10):1336-42.

7. Lagerros YT, Brandt L, Hedberg J, Sundbom M, Boden R. Suicide, Self-harm, and Depression After Gastric Bypass Surgery: A Nationwide Cohort Study. Annals of Surgery. 2017;265(2):235-43.

8. Tindle HA, Omalu B, Courcoulas A, Marcus M, Hammers J, Kuller LH. Risk of suicide after long-term follow-up from bariatric surgery. The American Journal of Medicine. 2010;123(11):1036-42.

9. Morgan DJ, Ho KM. Incidence and Risk Factors for Deliberate Self-harm, Mental Illness, and Suicide Following Bariatric Surgery: A State-wide Population-based Linked-data Cohort Study. Ann Surg. 2017;265(2):244-52.

10. Gribsholt SB, Thomsen RW, Svensson E, Richelsen B. Overall and cause-specific mortality after Roux-en-Y gastric bypass surgery: A nationwide cohort study. Surgery for Obesity and Related Diseases. 2017;13(4):581-7.

11. Bhatti JA, Nathens AB, Thiruchelvam D, Grantcharov T, Goldstein BI, Redelmeier DA. Self-harm Emergencies After Bariatric Surgery: A Population-Based Cohort Study. JAMA Surgery. 2015:1-7.
